# DNA repair enzymes in sunscreens and their impact on photoageing—A systematic review

**DOI:** 10.1111/phpp.12597

**Published:** 2020-08-27

**Authors:** Hanna Luze, Sebastian Philipp Nischwitz, Iris Zalaudek, Robert Müllegger, Lars Peter Kamolz

**Affiliations:** ^1^ COREMED—Cooperative Centre for Regenerative Medicine JOANNEUM RESEARCH Forschungsgesellschaft mbH Graz Austria; ^2^ Division of Plastic, Aesthetic and Reconstructive Surgery Department of Surgery Medical University of Graz Graz Austria; ^3^ Clinica Dermatologica Dipartimento di Scienze Mediche Chirurgiche e della Salute Università degli Studi di Trieste Trieste Italy; ^4^ Department of Dermatology and Venereology Federal Hospital Wiener Neustadt Wiener Neustadt Austria

**Keywords:** DNA repair enzymes, endonuclease, photoageing, photolyase, sunscreens

## Abstract

**Background:**

DNA damage is one of the main factors responsible for photoageing and is predominantly attributed to ultraviolet irradiation (UV‐R). Photoprotection by conventional sunscreens is exclusively prophylactic, and of no value, once DNA damage has occurred. As a result, the demand for DNA repair mechanisms inhibiting, reversing or delaying the pathologic events in UV‐exposed skin has sparked research on anti‐photoageing and strategies to improve the effect of conventional sunscreens. This review provides an overview of recent developments in DNA repair enzymes used in sunscreens and their impact on photoageing.

**Methods:**

A systematic review of the literature, up to March 2019, was conducted using the electronic databases, PubMed and Web of Science. Quality assessment was carried out using the Newcastle‐Ottawa scale (NOS) to ensure inclusion of adequate quality studies only (NOS > 5).

**Results:**

Out of the 352 publications, 52 were considered relevant to the key question and included in the present review. Two major enzymes were found to play a major role in DNA damage repair in sunscreens: photolyase and T4 endonuclease V. These enzymes are capable of identifying and removing UV‐R‐induced dimeric photoproducts. Clinical studies revealed that sunscreens with liposome‐encapsulated types of photolyase and/or T4 endonuclease V can enhance these repair mechanisms.

**Conclusion:**

There is a lack of randomized controlled trials demonstrating the efficacy of DNA repair enzymes on photoageing, or a superiority of sunscreens with DNA repair enzymes compared to conventional sunscreens. Further studies are mandatory to further reveal pathogenic factors of photoageing and possible therapeutic strategies against it.

## 1 INTRODUCTION

Intrinsic ageing of the skin is characterized by a slow and steady decrease of tissue function, for example tissue elasticity.^1^ Additional exposure of human skin to environmental factors can lead to an overlapping damage referred to as extrinsic ageing, photoageing or premature skin ageing.^1^ Photoageing describes changes in clinical, histological and functional characteristics of habitually UV‐exposed skin, which develop gradually over decades.^2^ Most of the unwanted skin changes during ageing such as wrinkle formation, laxity, leathery appearance or pigment changes are driven by photoageing. It also advances the loss of the skin's reparative and regenerative potential leading to higher vulnerability and an impaired wound healing response.^2^, ^3^, ^4^

DNA damage represents one of the main causative factors of photoageing and is predominantly attributable to ultraviolet‐radiation (UV‐R).^2^, ^5^, ^6^, ^7^ Strategies to prevent or minimize photoageing include protection against UV‐R and preservation of antioxidant homoeostasis.^3^ Recent studies investigated detrimental effects of UV‐R at the cellular and molecular levels to identify biological targets of UV‐R and the resulting cascade of impairment of cell functions and tissue degradation.^8^, ^9^ Both UV‐A and UV‐B radiations, including mutations of key regulator genes, have been found to cause DNA damage.^2^ Despite endogenous DNA repair mechanisms, DNA damage can persist and accumulate with chronic UV exposure, cumulatively leading to photoageing and skin cancer development.^2^, ^10^

An alarming increase of UV‐R due to the depletion of the stratospheric ozone layer and decrease in clouds and aerosols, especially in the northern mid‐latitudes, has been observed at the Earth's surface in the last two decades.^3^, ^11^ In addition, the exposure of human skin to environmental and artificial UV‐R has increased significantly due to changes in social behaviour, including an increase in average life expectancy.^3^, ^4^ As a result, there is an ever increasing demand for DNA repair treatments to inhibit, delay or reverse the process of these specific events.

Photoprotection by the application of conventional sunscreens is solely of prophylactic nature, and of no value, once DNA damage has occurred.^6^, ^12^ Whilst the existing literature has mainly addressed the impact of DNA repair enzymes on the UV‐associated development of malignancies, the present review aims to discuss recent findings regarding the biological process of photoageing and the role of DNA repair enzymes. An overview of the most relevant and currently available sunscreens containing DNA repair enzymes is provided below.

## 2 METHODS

We reviewed the medical literature in order to identify all studies investigating DNA repair enzymes in sunscreens, their ability to reduce or reverse DNA damage and their impact on photoageing. A systematic search of the literature published until March 2019 was conducted using the electronic databases PubMed and Web of Science as well as relevant reference lists.

**Figure 1:**
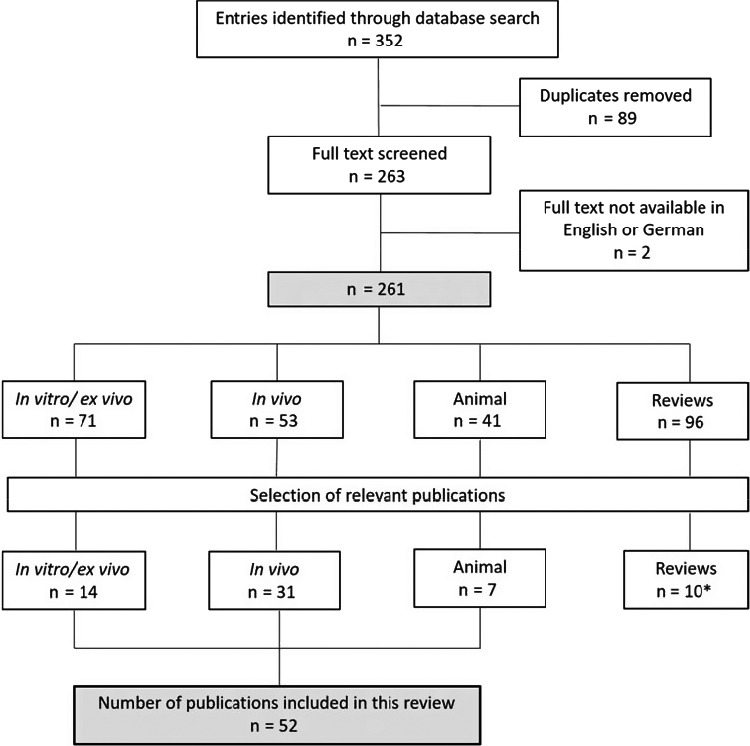
Flow diagram of study inclusion. *: Included reviews were used for basic information exclusively

Titles and abstracts were screened for the following key terms (variably combined): “DNA repair enzymes,” “sunscreen,” “photoageing,” “photoaging,” “skin‐ageing,” “treatment,” “photolyase,” “endonuclease.” Additionally, the reference lists of included articles were manually screened for further relevant publications. 352 publications evaluating the efficacy of DNA repair enzymes in sunscreens and their effect on photoageing were identified. If the abstract did not determine eligibility, full‐text evaluation was performed. Investigation of sunscreens containing DNA repair enzymes was determined as the fundamental inclusion criterion. After elimination of duplicates (n = 89), full‐text evaluation of the remaining publications was performed as shown in Figure 1. Only, the articles published in English or German language were included (exclusion of n = 2). Quality assessment was performed using the Newcastle‐Ottawa scale (NOS). Studies with NOS> 5 were considered of adequate quality, whereas studies NOS < 5 were deemed substandard. Data on study year, design and population were extracted from each study.

## 3 RESULTS AND DISCUSSION

Various mechanisms were described to be involved in photoageing. Among those, sun‐induced DNA changes represent one of the most potent causative factors. We present the impact of DNA repair enzymes in sunscreens on the reduction of DNA damage and prevention of photoageing. The 52 eligible studies were divided into 31 in vivo studies, 14 in vitro/ex vivo studies and 7 animal studies (mice). 10 pertinent reviews were exclusively used for basic information.

### 3.1 Sun‐induced DNA Damage

Recent research substantially promoted the understanding of mechanisms involved in photoageing. The principal pathogenic factor for photoageing is the presence of UV‐induced reactive oxygen species (ROS). Whilst low levels of ROS are continuously produced in the human body and have useful functions as important signalling molecules in physiological processes such as wound healing and repairing processes, there is evidence for a damaging effect of higher concentrations of ROS following UV‐R exposure.^13^, ^14^, ^15^ ROS activate cytoplasmic signal transduction pathways in resident dermal fibroblasts that are related to growth, differentiation, senescence and connective tissue degradation and therefore play a major role in photoageing.^4^ Permanent genetic alterations in the DNA caused by ROS, such as changes in gene expression pathways related to collagen degradation and elastin accumulation, are primarily repaired by the base excision repair (BER) system.^16^ 8‐hydroxy‐2'‐deoxyguanosine (8‐OHdG) is one of the predominant forms of free radical‐induced oxidative lesions in the DNA and, therefore, has widely been used as a biomarker for oxidative stress and carcinogenesis.^17^

Another way of DNA damage is caused by UV‐R directly; it leads to formation of dimeric photoproducts between adjacent pyrimidine bases.^16^ These photoproducts, namely cyclobutane pyrimidine dimers (CPD) and pyrimidine‐pyrimidone (6‐4) photoproducts (6‐4PPs), represent the most common UV‐induced DNA damage.^18^ In CPD, two adjacent thymine bases in the same DNA strand covalently connect to form a cyclobutane ring. In 6‐4PPs, in addition to the two bases being covalently linked, a complex chemical structure is formed, where oxygen and hydrogen atoms from one base migrate to another. Both CPD and 6‐4PP lead to mutagenesis, cell apoptosis and ultimately causing photoageing and skin cancer if not repaired by the body's own DNA repair mechanisms.^18^, ^19^

Several reports have shown a reduction in age‐related DNA repair capacity caused by an accumulation of processes including UV‐induced DNA damage, nucleotide excision repair (NER) and BER systems. This consequently contributes to the advancing of photoageing.^20^, ^21^, ^22^

The biochemical nature and the formation of DNA alterations highly depend on the wavelength of interfering photons.^16^ UV‐B radiation, which is the most energetic and mutagenic component of solar radiation, is directly absorbed by DNA and induces CPD and 6‐4PP development in particular.^16^ Although less energetic, thus of less damaging potential than UV‐B, the amount of UV‐A in natural sunlight is at least 20 times higher than of UV‐B. Hence, UV‐A is equally involved in the development of DNA lesions.^16^ The cytotoxic mechanism of UV‐A is strongly oxygen‐dependent and induces oxidative DNA lesions, mainly 8OHdG.^16^

Sunlight exposure also induces the formation of matrix metalloproteinase 1 (MMP‐1), an enzyme which is involved in the digestion of collagen 1.^23^ According to Fisher et al,^24^, ^25^ exposure to UV‐B not only leads to MMP‐1 induction, but within hours of irradiation, leads to upregulation of the transcription factors AP‐1 and NF‐κB, which act as stimulatory factors for MMP‐1 genes. Since the degradation of collagen is a hallmark of photoageing, MMP‐1 gene modulation represents another potential working point in reducing UV‐generated DNA damage.^23^, ^24^

Moreover, there is increasing evidence showing that in addition to UV‐R, infrared radiation (IR) may contribute to photoageing. IR accelerates the rate of wrinkle formation by upregulating skin angiogenesis.^26^ It has been demonstrated that both IR and UV‐R play an important role in skin angiogenesis by causing a dysregulation between the angiogenesis inducing vascular endothelial growth factor (VEGF) and the angiogenesis inhibiting thrombospondin 2 (TSP‐2).^27^ It has also been suggested that the IR‐induced skin angiogenesis may partially be caused by accumulation of heat in human skin.^27^ IR radiation further contributes to photoageing by gene activity modulation in mitochondria in several ways, for example an increased formation of mitochondrial ROS and the induction of MMPs.^26^

### 3.2 Mechanisms of sun‐induced photoageing

A central role in photoageing can be assumed for telomere‐based signalling.^28^ Telomere length is a molecular marker of a cell's age. Genomic instability due to telomere shortening has been linked to several ageing‐related conditions such as benign and malignant neoplasms and photo‐aged skin.^29^, ^30^, ^31^, ^32^, ^33^ Age‐related telomere shortening can be slowed down to some degree by telomerase, a ribonucleoprotein that adds DNA sequence repetitions in the telomere region.^34^, ^35^ However, most mammalian somatic cells do not express telomerase, which leads to the progressive and cumulative loss of chromosome‐protective sequences from chromosome ends.^36^ In vitro studies have linked accelerated telomere shortening to DNA‐damaging agents such as ROS.^37^, ^38^ In combination with UV‐induced damage to critical regulatory genes, this leads to the typical appearance of photoageing.^39^ Such mechanisms have been investigated in mouse models only.^32^, ^40^, ^41^ To understand the role of telomere shortening in human skin ageing, a respective model is required, since human telomeres are up to ten times longer and telomerase is less active as compared to mice.^32^, ^40^, ^41^

### 3.3 Current concepts of DNA repair enzymes in sunscreens

Blocking the transmission of UV‐R with sunscreens is a basic element for preventing photoageing.^1^ Several studies have demonstrated a protective effect of regular use of conventional sunscreens against the development of skin cancers.^42^, ^43^, ^44^ However, they do not have a repairing effect on skin cells, which have already been damaged by sun exposure.^6^ Conversely, “active photoprotection” is provided by sunscreens containing DNA repair enzymes and antioxidants in addition to the sun protection factor (SPF).^6^, ^45^ These compounds may overcome the current lack of solar radiation harm management by the dual mechanism of prevention and repair.^6^, ^45^ Previous insights into the molecular basis of photoageing have led to the development of certain enzymes that are capable of identifying and removing damaged cell DNA fragments. The two main representatives namely photolyase and T4 endonuclease V are discussed below.

### 3.4 Photolyase

Photolyase, a flavoenzyme containing the flavin adenine dinucleotide molecule, acts as a catalytic cofactor and repairs UV‐induced DNA damage of CPD and 6‐4PPs.^19^ Two different kinds of photolyases specifically repair CPD and 6‐4PP and thus are usually classified as CPD photolyases or 6‐4 photolyases, respectively, corresponding to their different substrates. The catalytic cofactor is conserved in the whole protein superfamily of photolyases and adopts a unique folded configuration at the active site that plays a critical functional role in DNA repair.^19^ Photolyase recognizes damaged thymine dimers and restores these lesions through direct absorption of blue light by the flavin adenine dinucleotide molecule or through energy transfer from an excited second chromophore, the antenna chromophore,^19^ which finally splits into individual pyrimidines and returns the electron back to the enzyme redox cofactor.^46^, ^47^

Application of photolyase is also associated with a reduction of MMP‐1 in epidermal and dermal compartments of the skin.^23^ Overexpression of MMP‐1 in human skin cells results in the destruction of collagen that plays a key role in photoageing.^23^ In addition, photolyase supports in‐cell regeneration that inhibits UV‐induced apoptosis and reduces skin inflammation caused by sunlight exposure by inhibition of the pro‐inflammatory cytokine interleukin 6 (IL‐6).^48^

Due to the extremely strong mutagenic potential of CPD (that exceeds the one of 6‐4PPs or other lesions, and their responsibility for the great majority of UV‐induced mutations), recent studies focussed primarily on CPD photolyase as a repair enzyme.^47^ Both in vitro and in vivo studies have demonstrated the beneficial properties of CPD photolyase in preventing photodamage.^12^, ^49^ Several clinical trials have been published on the use of a topical product containing liposome‐encapsulated CPD photolyase. It has been either used in patients without any skin lesions or as an adjuvant therapy in patients with actinic keratoses (AK), which represent in situ squamous cell carcinomas resulting from chronic sun exposure of the skin.^18^, ^50^ All human studies reported the use of sunscreens containing chemical UV filters combined with liposome‐encapsulated CPD photolyase.^12^, ^49^, ^51^ The liposome encapsulation provides a shuttle mechanism for the enzymes across the human stratum corneum and introduces biologically active proteins into the living epidermis.^52^ This mechanism may provide a new pathway for photoprotection against some forms of UV‐induced skin damage.^51^

Clinical trials in 2000^12^ and 2011^5^ found that the addition of CPD photolyase to conventional sunscreens contributes significantly to the reduction of UV‐R‐induced DNA damage and apoptosis when applied topically to human skin. The number of UV‐B radiation‐induced CPD decreased by 40%‐45%, which demonstrates the ability of photolyase to actively repair damage.^12^ However, these studies were conducted with small sample sizes, without a control group and mainly addressed carcinogenesis. Therefore, they are considered as “proof of concept” studies.

### 3.5 T4‐bacteriophage endonuclease V (T4 endonuclease V)

T4 endonuclease V is an enzyme, which was initially isolated from *Escherichia coli* infected with T4 bacteriophage. It initiates DNA repair at the site of UV‐induced CPD, which contributes to mutations that result in non‐melanoma skin cancers (NMSC) including the AK, if not repaired.^53^ The enzyme binds nontarget DNA in a salt‐dependent manner and screens the DNA by facilitated diffusion to locate its target site.^54^ Once UV‐damaged DNA is detected, cleavage occurs by two combined activities, the pyrimidine dimer‐DNA glycosylase activity and the apurinic‐apyrimidinic endonuclease activity.^55^ The efficacy and pace of naturally occurring DNA repair is enhanced approximately fourfold by the influence of T4 endonuclease V.^56^

In addition, the enzyme stimulates skin regeneration as well as skin reconstruction and prevents the destruction of extracellular matrix components, which contributes to the prevention of photoageing.^57^ For instance, MMP‐1 induction in human skin cells, by treatment with T4 endonuclease V, is reduced in the same way as by photolyase treatment, which results in the reduction of collagen‐destruction.^23^ Encapsulation of T4 endonuclease V into liposomes as delivery vehicles, termed “T4N5,” is required for an adequate penetration through the stratum corneum. Findings of a mouse study suggested that application of T4N5 to the skin may be a useful adjunct to sunscreens for prevention and reduction of deleterious local effects of UV‐R such as sunburn cell formation.^58^

### 3.6 Comparison of sunscreens alone vs sunscreens containing DNA repair enzymes

Advances in the understanding of skin biology have led to the development of a diversity of treatments aimed at preventing ageing and enabling skin rejuvenation. An ideal sunscreen should comprise different features: 1) protection against UV‐B radiation and long‐wavelength UV‐A radiation; 2) stability and safety of the filters; 3) ROS scavenging capability; and ideally 4) inclusion of enzymes contributing to cellular DNA repair.^6^

Recent irradiation studies indicated that the addition of DNA repair enzymes (CPD photolyase and T4 endonuclease V) to conventional sunscreens may reduce UV‐R‐induced molecular damage to exposed skin to a greater extent than conventional sunscreens alone.^59^ For example, in a clinical study by Carducci et al,^59^ a total of 28 AK patients were randomized to topically apply sunscreens with DNA repair enzymes (n = 14) or sunscreens alone (n = 14) for 6 months. The main outcome measures included hyperkeratosis, field cancerization and change in CPD levels in skin biopsies. It was shown that the CPD levels, compared to baseline values, decreased by 61% in patients who used sunscreens with DNA repair enzymes versus 35% in patients who used conventional sunscreens (*P* < .001), indicating their efficacy in reducing CPD formation.^59^

In another double‐blind study by Emanuele et al,^7^ the efficacy to reduce CPD formation of SPF 50 sunscreen with or without antioxidants (carnosine, atrazine, ergothioneine) and/or CPD photolyase was evaluated in biopsies of human skin obtained after irradiations. It was shown that the combination of topical antioxidants and CPD photolyase resulted in the highest reduction of CPD and free radical‐induced protein damage. The authors concluded that sunscreens containing antioxidants and photolyase are superior to conventional ones in reducing skin ageing, probably due to a synergistic effect.^7^ This study by Emanuele *et al* is the only clinical study that has investigated the effect of sunscreens containing DNA repair enzymes on photoageing so far. Details are further described in Table 1.

**Table 1:** Summary of the clinical study Emanuele et al^7^ on the impact of sunscreens containing DNA repair enzymes on photoageing

Cohort	Enzymes	Treatment period	Effects on photoageing	Dropout	Follow‐up	Adverse events
60 healthy Caucasian volunteers (30 males and 30 females), aged >18 and <65 y with Fitzpatrick skin type I‐II	Liposome‐encapsulated DNA repair enzyme complex (Photolyase, endonuclease, 8‐oxoguanine glycosylase)	8 consecutive days: exposure to ssUV‐R at 6 times MED. Between 30 and 45 min. before exposure: application of the test product. 24 h after the last exposure to ssUV‐R: biopsy harvesting for molecular analyses	A novel topical product (TPF50) consisting of three active ingredients (traditional sunscreen SPF50, DNA repair enzyme complex and antioxidant complex) showed best results in reducing formation of CPDs, PC and 8OHdG. TPF50 improves the genomic and proteomic integrity of skin cells after repeated exposure to UV‐R, ultimately reducing the risk of skin ageing.	No Dropout	No Follow‐up	No minor or major adverse events

Note

Abbreviations: 8OHdG, 8‐oxo‐7,8‐dihydro‐2‘deoxyguanosine; CPDs, Cyclobutane pyrimidine dimers; MED, Minimal erythema dose; PC, Protein carbonylation; SPF, Sun protection factor; ssUV‐R, Solar‐simulated ultraviolet‐radiation; UV‐R, Ultraviolet‐radiation.

Based on the existing evidence from similar studies, current research focuses on the development of new sunscreens and their enhanced protective effect. As an example, a sunscreen containing CPD photolyase (Eryfotona^®^ AK‐NMSC, Isdin SA) has recently been investigated. Clinical and histological studies demonstrated beneficial effects on field cancerization in AK patients, such as improvement in AK lesion count and extent of cancerization field.^18^, ^60^, ^61^ The effect could be transferred to photoageing as well, since DNA photodamage and ROS are the initial events in the development of AK alike.

Another recently developed product (Ateia^®^ Kwizda Pharma, Vienna, Austria) combines a conventional sunscreen with a patented ingredient formula, Nopasome^®^. Nopasome^®^ is a combination of liposome‐encapsulated CPD photolyase (Photosome^®^), T4 endonuclease V (Ultrasome^®^) and an extract of the nopal cactus. Another branded sunscreen product containing photolyase, Ladival^®^ med (STADA Arzneimittel, Bad Vilbel, Germany) is available with SPF 15 or 20.^62^ Ladival^®^ med 20 was tested in over 200 healthy subjects with good results regarding tolerance and efficacy.^62^ A summary of currently available sunscreens containing DNA repair enzymes with appropriate scientific background is listed in Table 2.

**Table 2:** Overview of sunscreens containing DNA repair enzymes currently available (alphabetic order)

Name	Company	SPF	DNA Repair	Relevant Studies included in this review
Ateia^®^	Kwizda Pharma GmbH, Vienna, Austria	50+ 50 30 25	Liposome‐encapsulated Photolyase, Endonuclease	Wolf et al ^51^
Eryfotona^®^ AK‐NMSC	Isdin, SA, Barcelona, Spain	100+	Liposome‐encapsulated Photolyase	Puviani et al ^18^
Heliocare 360° AK Fluid	Cantabria Labs, Madrid, Spain	100+	Liposome‐encapsulated Photolyase, Endonuclease, 8‐Oxoguanine Glycosylase	Stege et al ^12^ Yarosh et al ^52^
Ladival^®^ med	STADA Arzneimittel, Bad Vilbel, Germany	20 15	Liposome‐encapsulated Photolyase	Krutmann et al ^62^
Neova Active^®^ (SPF43) Neova Everyday^®^ (SPF44) Neova Silc Sheer^®^ 2.0 (SPF 40)	Pharma Cosmetics, Oradell, New Jersey, United States	43^a^ 44^a^ 40^a^	Liposome‐encapsulated Photolyase, Endonuclease	Puviani et al ^18^
Neova Smart Moisture^®^	Pharma Cosmetics, Oradell, New Jersey, United States	30^a^	Liposome‐encapsulated Photolyase	Puviani et al ^18^
Priori Tetra^®^	PRIORI Skincare, San Diego, California, United States	50^a^	Liposome‐encapsulated Photolyase, Endonuclease, 8‐Oxoguanine Glycosylase	Stege et al ^12^ Yarosh et al ^52^
Sesderma Repaskin^®^	Sesderma, Madrid, Spain	50 30	Liposome‐encapsulated Photolyase	Stege et al ^12^

Note

Abbreviations: SPF, Sun protection factor; UV‐B, Ultraviolet B.

^a^ American SPF (Currently, US products only measure SPF based on protection against UV‐B).

Due to the assumption that IR also contributes to photoageing, it is increasingly recommended that sunscreens should also offer IR protection. SkinMedica^®^ Total Defense + Repair (TD + R) (SkinMedica Inc, an Allergan Company) is a sunscreen, which combines broad spectrum UV protection with several antioxidants (SOL‐IR Advanced Antioxidant Complex^®^) that provide protection from IR radiation and promote skin repair.^26^ Preclinical studies have indicated that TD + R with a SPF 34 prevents the formation of UV‐induced sunburn cells and CPD.^26^ In addition, it prevents IR‐A‐triggered fragmentation of elastin fibres. It also preserves and/or improves the expression of extracellular matrix and downregulates MMP‐1 expression.^26^ A significant improvement in the appearance of lines and wrinkles was reported as early as week 2 in patients using TD + R SPF34.^26^

In summary, the current knowledge suggests that incorporation of DNA repair enzymes into conventional sunscreens provides a more efficient option for preventing UV‐R‐generated damage causing carcinogenesis and photoageing. Combining these with topically applied antioxidants offers a promising method to further amplify this effect. However, the evidence for these effects in humans, particularly regarding the prevention of skin ageing, is limited. In fact, only a single clinical study has demonstrated the effect of DNA repair enzymes in sunscreens on photoageing so far.^7^

## 4 LIMITATIONS

The limitations of the present review refer to the fact that topical liposomal DNA repair enzymes are known to be protective against UV‐induced skin cancer in humans, which does not necessarily explain their preventive effect on photoageing. Most of the included studies failed to clearly distinguish between the effects of DNA repair enzymes on carcinogenesis and photoageing. Thus, it is not possible so far to clearly transfer the results of improvement in carcinogenesis to therapy or prevention of photoageing. The small size of study cohorts further limits the relevance of their results. Clinical trials with a sufficient number of subjects, focusing specifically on anti‐photoageing effects, are warranted. Finally, our review is limited to articles retrieved from PubMed and Web of Science only with a slight possibility of missing other clinical trials.

## 5 CONCLUSION

Photoageing due to UV‐R causes undesirable changes in skin appearance. Its prevention with conventional sunscreens is inadequate because of their ineffectiveness to repair DNA damage. Key signatures of photodamage in the DNA represent a possible therapeutic target for studies of innovative therapeutic and preventive approaches to reduce photoageing.

So far, clinical studies have mainly addressed the association of DNA damage and cancerization. The efficacy of DNA repair enzymes and high‐protection UV filters in the treatment of actinic damage has also been well demonstrated. Due to the fact that UV‐R‐induced DNA damage is a main reason for photoageing alike, it can be assumed that the positive effects of DNA repair enzymes are transferrable to photoageing. However, controlled studies confirming this effect, as well as the superiority of sunscreens with DNA repair enzymes over conventional sunscreens, are still lacking. Future studies are essential for elucidating interactions between ROS, DNA damage, senescence, ageing and the role of telomeres in skin cells.

## CONFLICT OF INTEREST

The authors declare no conflict of interest.

## Data Availability

Data sharing was not applicable to this article as no data sets were generated or analysed during the current study.
